# Intraluminal Meckel's Duplication Cyst Causing Bowel Obstruction in an Infant: A Role for Laparotomy

**DOI:** 10.1155/2016/4717403

**Published:** 2016-10-13

**Authors:** Mitchell R. Ladd, Alejandro V. Garcia, Derek B. Allison, Jeffrey R. Lukish

**Affiliations:** ^1^Division of Pediatric Surgery, Department of General Surgery, Johns Hopkins Hospital, Baltimore, MD, USA; ^2^Department of Pathology, Johns Hopkins Hospital, Baltimore, MD, USA

## Abstract

This report describes a two-month-old girl who presented with signs and symptoms of a distal small bowel obstruction. She underwent an abdominal ultrasound that revealed a right lower quadrant cystic mass. A Technetium-99 scan revealed increased activity in the right lower quadrant consistent with a Meckel's diverticulum. Following a nondiagnostic laparoscopic evaluation, a laparotomy was performed to allow direct palpation of the small bowel and colon. Direct palpation of the ileum revealed a soft intraluminal mass at the ileocecal valve. The child underwent an ileocecectomy and anastomosis incorporating the intraluminal mass. Pathologic analysis revealed an intraluminal enteric duplication cyst containing ectopic gastric mucosa. This case represents the first report of such an entity in an infant. A discussion of the diagnostic and therapeutic aspects of the case and enteric duplication cysts is provided.

## 1. Introduction

Enteric duplication cysts are rare malformations with a reported incidence of 1 in 4500 with 80% of these diagnosed before the age of two [1]. Management of enteric duplication cysts involves surgical resection often performed laparoscopically [1]. We present a case of a duplication cyst containing gastric mucosa that was entirely intraluminal and therefore undetectable by laparoscopic evaluation. A laparotomy was necessary to locate the duplication cyst by manual palpation. There are no previous reports of a completely intraluminal ileal duplication cyst containing gastric mucosa.

## 2. Case Presentation

A two-month-old girl presented for evaluation for bilious emesis. She was a full-term female with an unremarkable perinatal history. At one month of age she developed pyloric stenosis and underwent an uneventful laparoscopic pyloromyotomy. She recovered well from this surgery and had been eating and gaining weight. On the day of presentation, she developed bilious emesis. She presented to the emergency room and underwent an abdominal X-ray that demonstrated small bowel dilation. Abdominal ultrasound was nondiagnostic but revealed a 4.8 × 2.8 cm fluid collection versus cyst to the right of midline. Her complete blood count revealed anemia with a hemoglobin count of 8.1 g/dL and her complete metabolic profile revealed a mild metabolic acidosis with a bicarbonate of 19 mEq/L.

**Figure 1:**
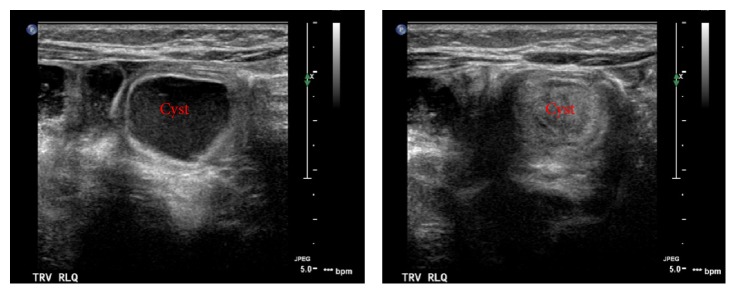
Abdominal ultrasound demonstrating a cystic structure in the right lower quadrant.

**Figure 2:**
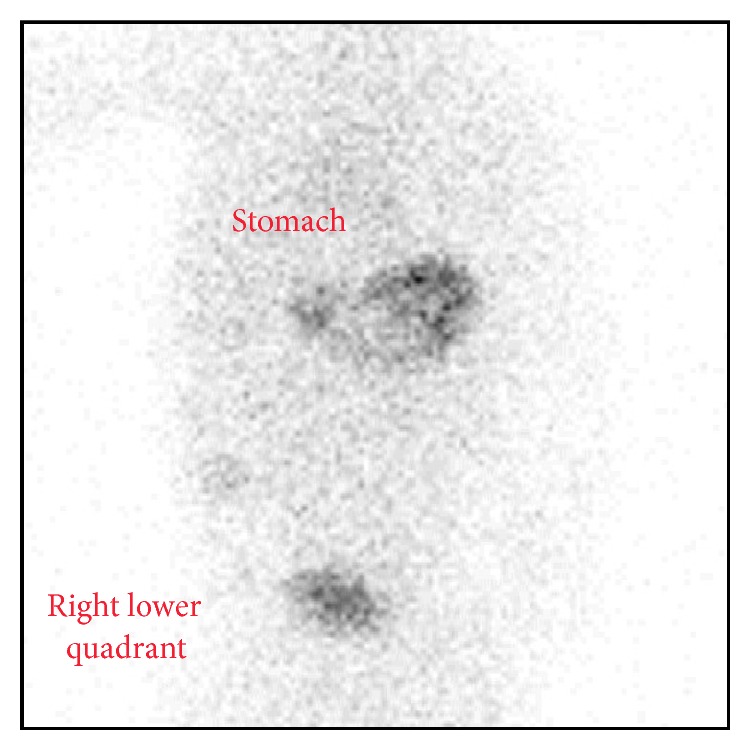
Technetium-99 scan demonstrating gastric mucosa in the stomach and in the right lower quadrant.

Following resuscitation, a limited upper gastrointestinal series was performed which showed normal intestinal rotation. An abdominal ultrasound was repeated which demonstrated a possible short-segment ileal-ileal intussusception with ileocecal inflammation. The previously identified fluid collection on prior ultrasound was noted to be in the area of the intussusception in the ileocecal region (Figure 1). She was admitted and underwent nasogastric decompression and nonoperative management. On hospital day 2, the baby was improving and tolerating feeds. However, she developed recurrent bilious emesis prior to discharge warranting further evaluation. A repeat complete upper gastrointestinal series with small bowel follow-through was performed that demonstrated the passage of oral contrast through mildly dilated loops of small bowel to the rectum but did not reveal an intestinal obstruction. The persistent and recurrent nature of this infant's symptoms, the unclear source of her anemia, and the abdominal fluid collection prompted our concern for Meckel's diverticulum. A Technetium-99 scan was performed which demonstrated increased activity in the right lower quadrant consistent with Meckel's diverticulum (Figure 2).

**Figure 3:**
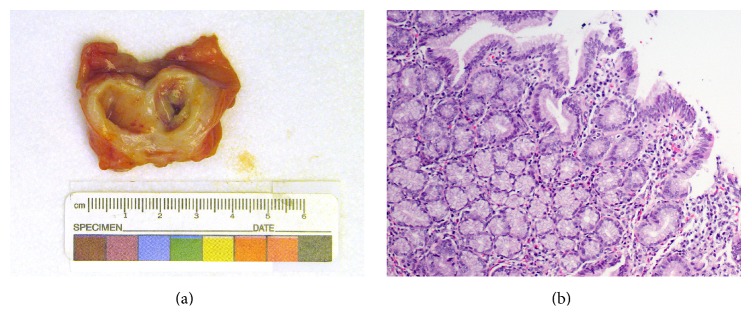
(a) Gross view demonstrating a completely intraluminal cyst. (b) Gastric, foveolar type mucosa is seen on the intraluminal surface of the cyst (H&E stain, 100x magnification).

Based on these findings, the patient was taken to the operating room on hospital day 3 for a diagnostic laparoscopy. The entire small bowel was investigated from the ligament of Treitz to the terminal ileum which did not reveal Meckel's diverticulum or any other abnormality. Consideration for an intraluminal lesion led to laparotomy. The abdomen was opened and the terminal ileum, cecum, and appendix were eviscerated. A spherical soft mass was palpated within the lumen of the terminal ileum. Resection of the terminal ileum and a portion of the cecum was performed incorporating the mass with a primary anastomosis. Postoperatively, the child did well and was discharged. Final pathology revealed an ileocecectomy specimen with a 3.2 cm segment of ileum, a 2.2 cm segment of cecum, and a 6 cm appendix. Adjacent to the ileocecal valve was a gastric-mucosa-lined intraluminal cyst (1.7 cm) with associated abscess formation. These findings, as demonstrated in Figure 3, were consistent with a completely intraluminal enteric cyst. The appendix and cecum were normal. In follow-up the child was doing well tolerating feeds and meeting all developmental milestones.

## 3. Discussion

Enteric duplication cysts can occur anywhere from oropharynx to rectum and have three major characteristics: (1) the presence of a smooth muscle coat, (2) an epithelial lining representing a type of intestinal mucosa, and (3) an anatomic association with a portion of the gastrointestinal tract (most commonly on the mesenteric aspect of the intestine) [1]. About 15–18% of duplication cysts occur in the mediastinum with 75% occurring in the abdominal cavity, of which ileal duplication cysts are the most common [1]. These duplications commonly share a common wall with the main alimentary tract lumen but can also be isolated in the mesentery. Duplication cysts typically share the same blood supply as the underlying intestine which precludes cystectomy and often requires resection of the associated bowel [1, 2]. Ectopic gastrointestinal epithelium can be present ranging from gastric to rectal epithelium. The type of epithelium present usually corresponds to the adjacent associated bowel. Approximately 20–30% of gastrointestinal duplications contain ectopic gastric mucosa which are ultimately diagnosed either at the time of surgery or preoperatively with a Technetium-99 scan [1, 3–9]. Patients with such duplication cysts typically present with abdominal pain due to ulcer formation from acid-secreting mucosa, obstruction secondary to the cyst itself, or intussusception with the cyst serving as a lead point resulting in gastrointestinal bleeding or even perforation [1–3].

Enteric duplication cysts can be classified into three subtypes: saccular (spherical), tubular, and small intramural [2]. Saccular is the most common type which usually does not communicate with the lumen and is often asymptomatic. Tubular cysts tend to occur more commonly in the colon and are often associated with genitourinary defects [2]. Small intramural cysts often occur near or at the ileocecal valve and protrude into the bowel lumen often causing bowel obstruction [2].

Here we present a rare case of a completely intraluminal duplication cyst similar to an intramural cyst. This duplication cyst was in close association with the ileocecal valve, resulting in obstructive symptoms and signs of intussusception. The diagnosis in this case was further confounded by the recent pyloromyotomy and potential adhesive small bowel obstruction. When she presented with emesis, there was initial concern that the pyloromyotomy had been inadequate. However, the bilious nature of the emesis suggested a more distal bowel obstruction. Our differential diagnoses included malrotation, volvulus, intussusception, constipation, or a distal intestinal obstruction from adhesions from prior surgery. The initial workup was most consistent with an ileoileal intussusception. We manage this nonoperatively with nasogastric decompression and resuscitation. If the child's condition deteriorates or the child develops a complete bowel obstruction operative intervention may be necessary, but surgical intervention is uncommon [10, 11]. It is also rare for an infant to have a pathologic lead point related to intussusception. The suggestion of an intussusception on ultrasound in the setting of a right lower quadrant cyst prompted the Technetium-99 scan to rule out Meckel's diverticulum.

The minimally invasive approach to intussusception and Meckel's diverticulum is logical and well described [10–13]. The main dilemma in this case was the operative findings or lack thereof. Typically, Meckel's diverticulum can be located via diagnostic laparoscopy and resected and intussusception can be reduced using minimally invasive techniques. In this case, the external appearance of the intestine was completely normal. Based on the Technetium scan our suspicion for an intraluminal lesion was high, and therefore we turned to an open operation in order to palpate the intestine. Only with direct palpation was the hidden cystic lesion located. The lesion was 1.7 cm and intraluminal and therefore could have easily been missed.

In conclusion, we present a rare case of an intraluminal enteric duplication cyst containing gastric mucosa causing intussusception in an infant. To our knowledge, this is the first report of such a case. This case highlights the importance of considering both Meckel's diverticulum and an enteric duplication cyst in the differential of causes for intussusception in children. If the Meckel scan is positive, we recommend the need for direct palpation of the intestine if initial laparoscopy is negative to rule out a small intraluminal anomaly.
